# Impact of Ultrasonic-Assisted Preparation of Water Caltrop Starch–Lipid Complex: Structural and Physicochemical Properties

**DOI:** 10.3390/foods14020240

**Published:** 2025-01-14

**Authors:** Kuan-Wei Huang, Lih-Shiuh Lai

**Affiliations:** Department of Food Science and Biotechnology, National Chung Hsing University, 145 Xingda Road, Taichung 40227, Taiwan; jason123314@gmail.com

**Keywords:** ultrasound intensity, starch-lipid complex, structural characteristics, viscosity

## Abstract

This study investigates the effect of ultrasonic-assisted preparation on the structural and physicochemical properties of water caltrop starch-palmitic acid complexes as a function of ultrasound intensity and treatment time. All samples exhibited the characteristic birefringence of starch-lipid complexes under the polarized microscope, and flake-like and irregular structure under scanning electron microscope (SEM), indicating the formation of complexes through ultrasonic-assisted preparation. X-ray diffraction pattern further confirmed the transition from the original A-type structure for native starch to V-type structure for starch-lipid complexes, and the relative crystallinity of starch-lipid complexes increased as the ultrasound intensity and treatment time increased. Attenuated total reflectance-Fourier-transform infrared spectroscopy (ATR-FTIR) analysis indicated a decreasing trend in absorbance ratio at wavenumber of 1022 cm^−1^/995 cm^−1^, suggesting that the increase in the complex promoted the self-assembly within the short-range ordered structure, leading to the formation of bonds between the complexes. However, rapid-visco analysis (RVA) demonstrated that the viscosity generally decreased as the ultrasound intensity and treatment time increased, possibly due to the reduction in molecular weight by ultrasound. Differential scanning calorimetric (DSC) analysis revealed that the control starch-lipid complex without ultrasound treatment (US-0-0) exhibited two distinct endothermic peaks above 90 °C, representing Type I (95–105 °C) and Type II (110–120 °C) V-type complexes. However, ultrasound-treated samples showed only one peak around 95–105 °C and increased enthalpy (∆H), which was likely due to the breakdown of amylose and amylopectin, leading to more complex formation with palmitic acid, while the resulting shorter chains in the ultrasound-modified sample favor the formation of Type I complexes.

## 1. Introduction

In recent year, people are paying more attention to their diets, focusing not only on the taste of food but also on its effect on health. Type V resistant starch, also known as starch-lipid complexes, has been found to have a positive effect on gut health by promoting intestinal motility and regulating the gut microbiota [1,2,3]. Including starch-lipid complex in the diet offers a promising approach to enhancing gut health. Moreover, starch-lipid complexes have shown commercial promise as natural emulsifiers, and may impact the functionality of processed foods [4,5,6]. The V-amylose structure is a broad term that refers to a variety of crystalline amyloses, in which single helices are co-crystallized with inclusion substances like water, iodine, DMSO, fatty acids, alcohols, and others [7,8]. During the complex formation process, amylose must be solubilized in an aqueous solvent or heated to a high temperature to disrupt the native helical segments. In addition, in some allomorphs of amylose-lipid complexes with less compact structure, guest molecules and water molecules may be located not only inside but also between single helices [9,10,11]. The formation of starch-lipid complexes involves mainly amylose, but also possibly long side chains of amylopectin [3,12]. Furthermore, the structural form of starch-lipid complexes is influenced by the type and concentration of fatty acid, crystallization temperature and solvent composition, which affect the helicity of amylose and the resulting allomorphs [9]. Though short-chain fatty acids with carbon chains ranging from C3 to C5 generally tend to induce the formation of 7-fold V-amylose helical structures, and long-chain fatty acids, such as palmitic acid (C_16:0_), are more likely to promote the folding of amylose into 6-fold V-amylose helical structures, different types of helices can also be obtained with the same fatty acid, depending on the crystallization conditions [9]. During complexation, the alkyl moiety of the fatty acid is encapsulated within the 6-fold or 7-fold V-amylose helices, while its polar carboxyl group end is located on the exterior of the helix. These helical structures subsequently aggregate to form crystalline assemblies. Putseys, Lamberts and Delcour [8] noted that the degree of glucose polymerization required for amylose to complex with fatty acids varies depending on the chain length of fatty acids. In the case of palmitic acid, at least 30 to 40 glucose residues are needed for complex formation, while lauric acid can complex with as few as 20 to 30 glucose residues. In addition to chain length of amylose or long side chains of amylopectin, and the size of the starch helical cavity [9], several factors affect the formation of the complexes, including the carbon chains and saturation of fatty acids, dispersion and solubility of starch molecules [13,14,15], as well as the specific processing conditions used for preparation [16,17]. Understanding the interaction between starch-lipid complexes and fatty acids, as well as the factors that affect their formation, can significantly enhance the efficiency of complexation, allowing for the preparation of starch-lipid complexes with desirable characteristics.

Ultrasonic-assisted technology, as a non-thermal physical modification technique, plays a significant role in food processing and manufacturing due to its advantages of short processing time, high efficiency, and low pollution. The cavitation effect, shock waves, and mechanical forces generated by ultrasonic treatment can help break down and disperse samples more evenly, increasing production efficiency while reducing energy waste [18,19]. The phenomenon has broad applications in fields such as ultrasonic cleaning, medical ultrasound therapy, and underwater sonar [2,20,21]. In recent years, there has been limited research on the application of ultrasonic technology in preparing starch-lipid complexes for the food industry [22,23]. The ultrasonically-induced high dispersion may enhance the formation between main starch molecules and guest fatty acid, in addition, the cavitation effects of ultrasound may break down starch molecules, thereby influencing their inclusion capability with fatty acid molecules [24,25]. Zhang, Mi, Gao, Wu, Yuan, Cui, Dai and Liu [2] employed ultrasonic treatment prior to and after incorporating fatty acid and starch, and found that applying ultrasonic treatment prior to mixing these two materials led to complex formation with superior physical properties, such as crystallinity and thermal characteristics. Existing studies have explored various combinations of starches and fatty acids, as well as different ultrasonic operating conditions, such as amplitude and combined frequencies [1,2,18,20,22,24,25,26,27]. However, the role of ultrasound varies, and the mechanisms by which ultrasonic treatment influences the formation of starch-lipid complexes and the correlations between physicochemical properties and these complexes remain inadequately addressed.

Water caltrop (*Trapa taiwanensis* Nakai) is a common crop in southern Taiwan, characterized by its horned shape, which can have either two or four spikes. Water caltrop, a traditional food frequently seen in Taiwanese markets, is known for its high nutritional value and significant commercial potential [28,29,30,31]. Among various starchy crops, water caltrop has been shown to have several unique characteristics, including a much higher resistant starch content [29,30,31]. However, research about water caltrop starch remains relatively limited. Therefore, the objective of this study is to utilize ultrasonic-assisted technology to prepare starch-lipid complexes and assess whether this method can enhance the complexation efficiency of water caltrop starch-lipid complexes. The aim is to develop a more efficient process for producing these complexes. Additionally, by applying various ultrasound operating parameters, the study seeks to identify the optimal conditions for preparing water caltrop starch-lipid complexes using ultrasonic-assisted technology. A comprehensive analysis will also be conducted on the structure and physicochemical properties of the prepared starch-lipid complexes.

## 2. Materials and Methods

### 2.1. Material

The water caltrop was purchased from Xiaying District, Tainan City, Taiwan. Palmitic acid (C_16:0_) was purchased from the Choneye Pure Chemicals (Taipei, Taiwan), with a purity level of Extra Pure grade. The amylose/amylopectin assay kit was purchased from the Megazyme International Ireland, Co. (Wicklow, Ireland). All other chemicals used were of analytical grade.

### 2.2. Starch Isolation

Starch isolation procedures were essentially followed the method of Chung and Lai [29]. The fresh water caltrop kernels were chopped and placed into a blender with twice the volume of pure water, homogenized, and then filtered. To remove pigments and proteins, 0.1% (*w*/*v*) NaOH was added, and the sediment was repeatedly washed with pure water until the pH of the starch supernatant reached to 7. The sample was then dried in an oven at 40 °C till the moisture content was less than 10%, ground, sieved through a 100-mesh sieve, and stored at room temperature for further analysis. The chemical compositions of the isolated water caltrop starch were determined following the Association of Analytical Chemists (AOAC) standard methods. The crude protein, crude lipid, crude fiber, and ash contents were found to be 0.36%, 0.15%, 0.00%, and 0.16%, respectively, implying high purity of the isolated starch. The amylose content of water caltrop starch was determined using the amylose/amylopectin assay kit from Megazyme International Ireland, Co. (Wicklow, Ireland), and was found to be 20.53%.

### 2.3. Preparation of Ultrasonic-Assisted Starch Lipid Complex

Per 15 g of water caltrop starch was mixed with 300 mL of double-distilled (DD) water. The starch solution was gelatinized by heating and stirring in a water bath at 95 °C for 35 min. The gelatinized starch paste was then placed in an ultrasonic machine (Q700 Sonicator, QSONICA, Newtown, CT, USA) with an ultrasonic frequency of 20 kHz for ultrasound treatment at amplitudes of 30% and 50% for 5, 10, and 20 minutes, respectively. A circulating water-cooling system (DC30-K10, Thermo Fisher, Waltham, MA, USA) was used to maintain the treatment temperature at 25 °C, and pulsed ultrasonic treatment were applied with a 5-s on and 5-s off cycle to prevent overheating from the set temperature. The ultrasonic probe used had a specification of 19 mm. After ultrasonic treatment, samples were reheated to 95 °C on the heating stirrer, and 1.5 g of palmitic acid pre-dissolved in 15 mL ethanol was added for complexing reaction with starch for 1 h. The resulting complex was precipitated and washed with ethanol to remove free fatty acids, followed by centrifugation at 12700× *g* for 15 min using a centrifuge (CR22N, Hitachi Instruments Inc., Tokyo, Japan). The supernatant was removed, and the sample was dried at 40 °C, ground and sieved for further analysis. Sample codes for the starch-lipid complex samples are denoted as US-x-y, where x represents the ultrasonic amplitude and y represents the ultrasonic treatment time, respectively. The starch-lipid complex without ultrasonic treatment (US-0-0) is used alongside native starch and gelatinized starch as the control group.

### 2.4. Polarized Light Microscopy

The birefringence of the samples were examined using an optical microscope (BX41, Olympus) with a polarizing filter (BX-POL, Olympus, Tokyo, Japan) placed above the light source of the microscope. A few complex samples were placed on a microscope slide, added with a drop of 50% glycerol, then covered with a coverslip and examined under microscope. Microscopic images were recorded by connecting the microscope to a microphotography system using a camera adapter (U-CMAD3, Olympus, Tokyo, Japan) and a digital camera (MC4K, Ostec Electronic Technology Co., Ltd., Guangzhou, China).

### 2.5. Scanning Electron Microscopy (SEM)

The microstructure of the samples was observed using scanning electron microscope (JSM-7800F, JEOL, Tokyo, Japan). The samples were firmly adhered to double-sided tapes followed by thin platinum coating using a metal ion coater (JEC-3000FC, JEOL, Tokyo, Japan) under 10 mA for 30 s. Samples were then examined at an accelerating voltage of 3.0 kV.

### 2.6. X-Ray Diffraction (XRD)

The long-range ordered structure of the samples was analyzed using X-ray diffractometer (X’Pert Pro MPD, Panalytical, Almelo, Netherland). Approximately 0.2 g of the sample was placed in a glass desiccator with a saturated sodium chloride solution (relative humidity = 75%) at room temperature for one week to equilibrate the structure. The experimental conditions for the analysis included the use of a copper target (Cu K_α_, wavelength = 0.15418 nm) as the radiation source, with a scan angle (2θ) ranging from 5° to 50° and a scan step of 0.02°, operated at a voltage of 45 kV and a current of 40 mA. The relative crystallinity of the complex was calculated using PeakFit software (V4.12, 1995, Jandel Scientific Software, AISN Software Inc., Erkrath, Germany) with the following formula:

(1) $$\mathrm{Relative}\;\mathrm{Crystallinity}\;(\mathrm{RC})=\frac{I_{c}}{I_{c}+I_{a}}\times \;100\%$$

where I_c_ and I_a_ represent the integrated intensity of the crystalline region and the amorphous region, respectively.

### 2.7. Attenuated Total Reflectance-Fourier-Transform Infrared Spectroscopy (ATR-FTIR)

The short-range order of the samples was analyzed following the procedure outlined by Tsai and Lai [30], using Fourier Transform Infrared (FTIR) Spectroscopy (Vertex 70V FTIR Spectrometer with Hyperion 3000 FTIR Microscope and MCT detector, Bruker Scientific LLC, Billerica, MA, USA) equipped with an attenuated total reflection (ATR) accessory. Sample was dried in an oven at 50 °C for 24 h prior to analysis. The spectral range was set from wavenumber 4000 cm^−1^ to 650 cm^−1^, with a resolution of 4 cm^−1^ and 64 scans. The absorbance ratios under wavenumbers of 1047 cm^−1^/1022 cm^−1^ and 1022 cm^−1^/995 cm^−1^ were calculated.

### 2.8. Rapid Viscosity Analysis (RVA)

The continuous heating/cooling viscosity profiles of the samples were analyzed using a Rapid-Visco Analyzer (RVA-Ezi, Newport Scientific Pty Limited, Warriewood, NSW, Australia), with a 6% sample solution placed in the RVA aluminum cylindrical sample chamber. The experimental conditions were set as follows: the starting temperature was 50 °C, with a stirring speed of 960 rpm for 10 s, followed by a reduction in speed to 160 rpm for 50 s. Over the next 3.5 min, the temperature was increased at a rate of 12.86 °C per minute to reach 95 °C, where it was held for 3 min. The temperature was then lowered back to 50 °C over the next 3.5 min and held at 50 °C for another 3 min. The viscosity profiles as a function of time and temperature were recorded in centipoise (cP).

### 2.9. Thermal Properties

Thermal properties of the samples were measured by using a differential scanning calorimetry (DSC) [18]. A total of 3.2 mg (dry basis) of the sample and 12.8 mg of distilled water were added to a 40 µL aluminum pan (ME-2277331, Mettler Toledo, Greifensee, Switzerland) and placed in a refrigerator at 4 °C for 24 h for moisture equilibration. The sample pan, along with a blank aluminum pan, was then placed in a differential scanning calorimeter (DSC 1 STAR system, Mettler Toledo, Switzerland) for measurement. The conditions were set as heating from 30 °C to 130 °C at a rate of 10 °C per minute. The onset temperature (T_o_), peak temperature (T_p_), end set temperature (T_e_) and enthalpy (ΔH) of phase transitions of the samples were recorded.

### 2.10. Complex Index (CI)

The complex index of the samples was determined essentially according to the method of Wu, et al. [32]. 0.25 g of the sample was accurately weighed and dispersed in 40 mL of distilled water. The thoroughly mixed dispersion was then heated in a water bath at 100 °C for 10 min and centrifuged at 5000× *g* for 15 min. 0.3 mL of the supernatant was then mixed with 3 mL of iodine solution (containing 2.0% KI and 1.3% I_2_), and the absorbance at 690 nm was measured using Microplate Full-Spectrum Absorption Spectrometer (SPECTROstar Nano, BMG LABTECH, Ortenberg, Germany). Gelatinized starch without complexation reaction was taken as the reference. The complex index was calculated with the following equation:

(2) $$\mathrm{Complex}\;\mathrm{index}\;(\mathrm{CI})\;\%=\frac{A_{r}-A_{s}}{A_{r}}\times \;100\%$$

where $A_{r}$ and $A_{s}$ represent the absorbance of the reference (gelatinized starch) and the complex sample at 690 nm, respectively.

### 2.11. Statistical Analysis

Variance analysis was performed by using SPSS (Statistical Product and Service Solution, version 19, IBM, Armonk, NY, USA). Duncan’s New Multiple Range Test was employed to compare the differences between means, and *p* < 0.05 denotes a significant difference between means statistically.

## 3. Results and Discussion

### 3.1. Polarized Light and Scanning Electron Microscopic Observation

**Figure 1:**
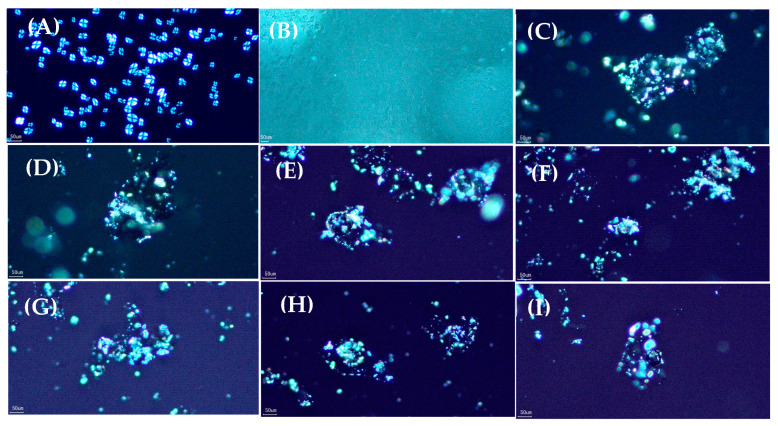
Polarized light microscopy images of native, gelatinized water caltrop starch and water caltrop starch-lipid complexes with various ultrasonic-assisted treatments. (**A**) Native water caltrop starch, (**B**) Gelatinized water caltrop starch, (**C**) US-0-0, (**D**) US-30-5, (**E**) US-30-10, (**F**) US-30-20, (**G**) US-50-5, (**H**) US-50-10, and (**I**) US-50-20.

The multiscale structures of the samples were analyzed using polarized light microscopy and scanning electron microscopy (SEM), respectively. Figure 1 shows the images of samples from polarized light microscopy. Native water caltrop starch displays a well-defined birefringence pattern, indicating the structural integrity. After gelatinization, the birefringence disappears due to the destabilization of the starch granules under high temperature and excess water, leading to water absorption, swelling, and eventual loss of the ordered crystalline structure. Native water caltrop starch contains approximately 0.15% endogenous lipids, which is considerably lower than the lipid content in cereal starches, such as 0.8% in regular corn starch and 0.9% in wheat starch [33]. Moreover, only free fatty acids, mono-acylglycerols, and partial diacylglycerols can complex with amylose, but not triglycerols/triglycerides [33]. As a result, the formation of complexes with endogenous lipids in water caltrop starch appears to be limited, as indicated by the absence of birefringence under polarized light microscopy. In contrast, with the addition of palmitic acids, all samples with and without ultrasonic treatment show pronounced polarized features of a V-type crystalline structure, qualitatively confirming the effectiveness of ultrasonic process for the formation of self-assembled starch-lipid complexes [13,34]. It should be mentioned here that though ethanol may form V6-type complexes with amylose, the effectiveness depends on the amount of ethanol and the processing temperature [35]. In this study, ethanol was used to pre-dissolve palmitic acid for complex formation, and to wash off the free fatty acids after complexation reaction. In the total reaction mixture, the amount of ethanol used to pre-dissolve palmitic acid is significantly lower than the amount required for complexation. During washing-off the free fatty acid step, the temperature has already been cooled down to room temperature. Therefore, the influence of ethanol on the overall yield in complexes should be minimal.

**Figure 2:**
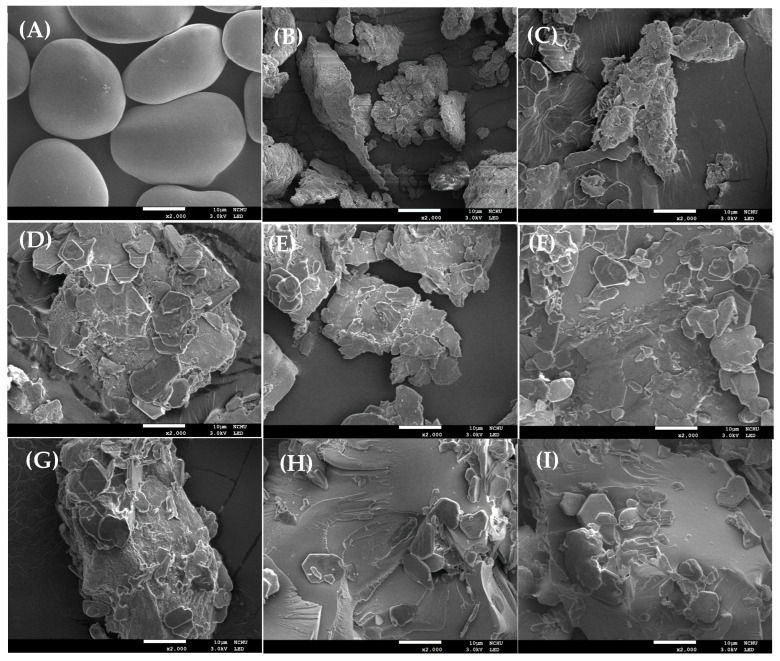
Scanning electron microscopy images of native, gelatinized water caltrop starch and water caltrop starch-lipid complexes with various ultrasonic-assisted treatments. (**A**) Native water caltrop starch, (**B**) Gelatinized water caltrop starch, (**C**) US-0-0, (**D**) US-30-5, (**E**) US-30-10, (**F**) US-30-20, (**G**) US-50-5, (**H**) US-50-10, and (**I**) US-50-20.

Figure 2 shows the SEM images of native, gelatinized water caltrop starch, and water caltrop starch-lipid complexes with and without various ultrasonic-assisted treatments. The native water caltrop starch exhibits smooth and intact granules, and is consistent with the typical morphology of native water caltrop starch [29]. After gelatinization, drying and grinding procedure, the granule structures of starch are disrupted, resulting in fragment-like shapes. With the addition of fatty acid, significant changes on surface morphology of gelatinized starch are induced. All starch-lipid complex samples exhibit irregular, layered flake-like structures, likely resulted from the self-assembly of starch and fatty acid molecules during the complexation process. Somewhat hexagonal flat particles are also observed, possibly due to the residual un-complexed fatty acid. Tang, Liang, Ren, Raza and Ma [25] and Wang, et al. [36] report that the starch-fatty acid complexes exhibit uneven, block-like structures. Li, et al. [37] observed a membrane-like material covering the complex surfaces, suggesting the formation of stacked lamellar structures by starch-lipid complexes.

### 3.2. Long-Range Ordered Molecular Structure by XRD Analysis

**Figure 3:**
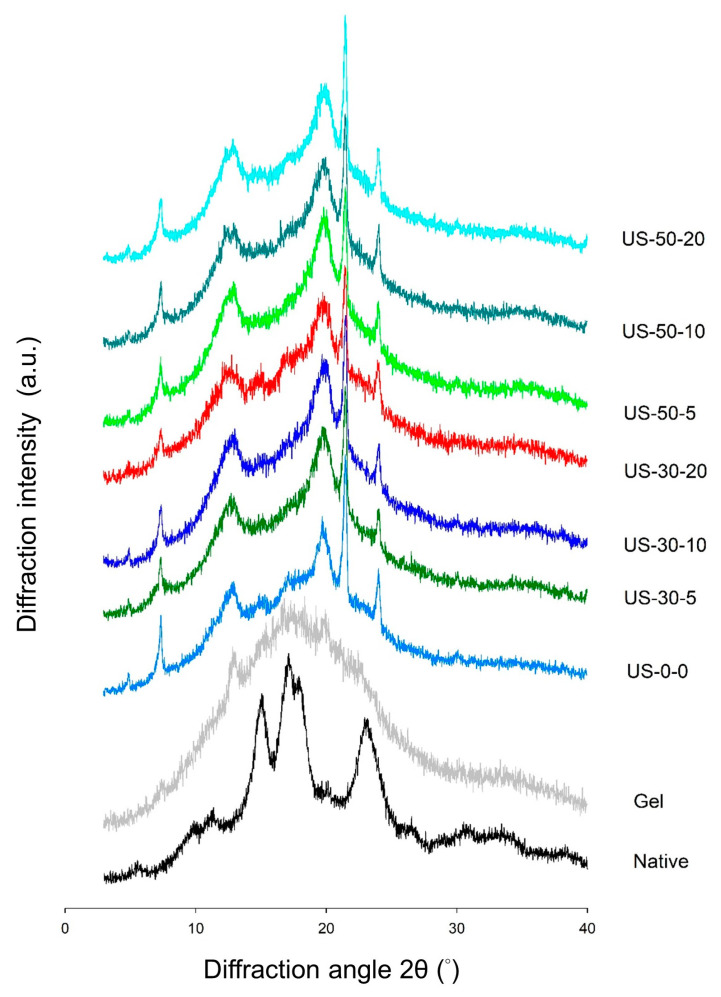
XRD profiles of native, gelatinized water caltrop starch and ultrasonic-assisted water caltrop starch-lipid complexes. “Native” denotes the native water caltrop starch, “Gelatinized Starch” denotes the gelatinized water caltrop starch. “US-0-0” denotes the starch-lipid complex without ultrasonic treatment. “US-30-5”, “US-30-10” and “US-30-20” denote samples treated with 30% ultrasonic amplitude for 5, 10, and 20 min, respectively. “US-50-5”, “US-50-10”, and “US-50-20” denote samples treated with 50% ultrasonic amplitude for 5, 10, and 20 min, respectively.

X-ray diffraction (XRD) can be employed to examine the long-range order of starch samples, which pertains to the packing of double helices into ordered crystalline structures, defined by the crystalline type and the degree of crystallinity. The XRD profiles of native starch, gelatinized starch, and starch-lipid complexes are shown in Figure 3. Native water caltrop starch exhibited diffraction peaks at 9.9°, 11°, 15°, 17°, 18°, and 22°, indicating a typical A-type crystalline structure. After gelatinization, the original A-type crystalline structure of starch is degraded by sufficient heat and excess water, and the formation of complexes with endogenous lipids is limited, resulting in absence of distinct characteristic diffraction peaks. With the addition of fatty acid, significant changes on XRD profiles of gelatinized starch are induced. For all complex samples prepared with and without ultrasonic treatment, distinct diffraction peaks at 7.5°, 13°, and 20° are observed, which are indicative of the V-type crystalline structure by the presence of amylose-lipid complexes. Though most of the free fatty acids has been washed off after complexation reaction, the thin peaks appearing at 21.4° and 24° reveal the presence of residual un-complexed palmitic acid [38], and is consistent with the SEM results shown in Figure 2. Furthermore, as shown in Table 1, the relative crystallinity of native water caltrop starch is around 47%, which is comparable with previous research of water caltrop starch [29], but a bit high as compared to other starch sources. Water caltrop starch has been shown to have higher gelatinization temperature and resistant starch content than those commonly used starch sources, implying the well-ordered internal structure of granules [29,30,31]. The high crystallinity of native water caltrop starch also indicates a well-organized internal structure within the granules. After gelatinization, the relative crystallinity of water caltrop starch decreases significantly to 3.21%. Though by definition, gelatinized starch has lost its crystalline structure, the weak crystallinity of gelatinized water caltrop starch is possibly due to the retrogradation upon cooling to room temperature, followed by subsequent drying and aging. For the starch-fatty acid complex samples, the relative crystallinity of the control group (US-0-0) increased to 21.30%, suggesting that the formation of V-type crystalline structures contributes to the increase in crystallinity. Ultrasonic pretreatment further increases the relative crystallinity of the complex samples as compared to the control group without ultrasonic treatment, and is more pronounced with increasing ultrasound duration, which is consistent with the findings of Tang, Liang, Ren, Raza and Ma [25]. In comparison to the treatment time, increasing ultrasound amplitude impacts less on the relative crystallinity of starch-lipid complexes. The cavitation effects of ultrasound are random so one can expect a polydisperse distribution of various branched maltodextrins. The residual branching point will probably hinder the ability to form stable single helices with lipids and crystallize. However, the prolonged ultrasound treatment may also lead to significant breakdown of amylose and amylopectin and increased release of apparent amylose from long segments of amylopectin, which promoting the formation of more starch-lipid complexes [18]. Although the literature generally suggests that the amount of complexes increases with the degree of polymerization (DP) of amylose, Godet, et al. [39] find the inclusion of the fatty acid may not be complete, and possibly more frequent in complexes prepared with high DP of amylose, where amylose chains may be involved in several crystalline areas. No and Shin [40] mention that the DP of amylopectin side chain typically ranges from 12 to 37. Considering that some degraded amylopectin chains may have too small DP to participate in complex formation, there are still longer degraded amylopectin chains that may contribute to the formation of complexes. These complexes could aggregate, forming crystalline structures and thereby increasing the overall crystallinity. Similar finding has also been reported by Hao, Xu, Yu, Han, Gu, Wang, Li, Zhang, Deng and Xiao [18].

### 3.3. Short-Range Ordered Molecular Structure by ATR-FTIR Analysis

**Figure 4:**
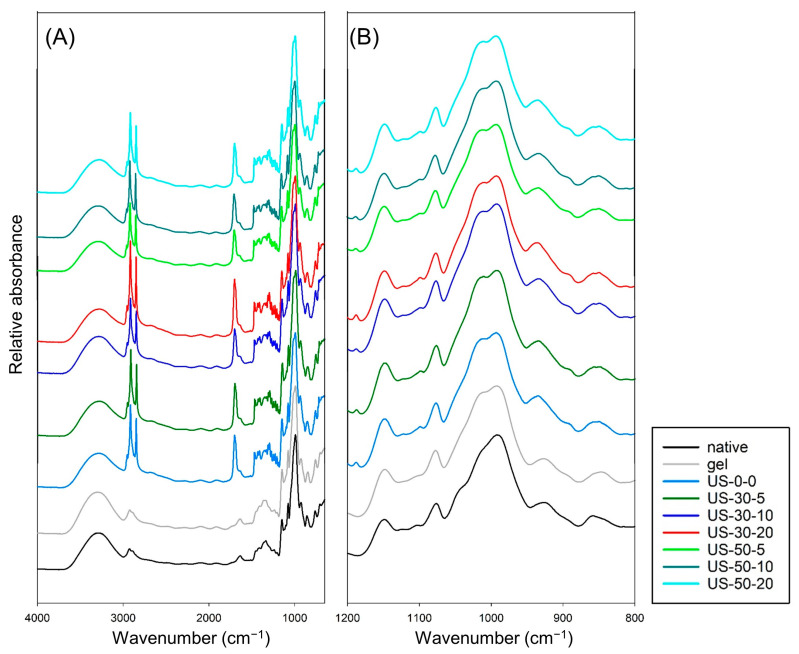
ATR−FTIR spectra of native, gelatinized water caltrop starch, and ultrasonic-assisted water caltrop starch-lipid complexes in the range of (**A**) 4000–650 cm^−1^ and (**B**) 1200–800 cm^−1^. Sample codes are the same as described in Figure 3.

The short-range ordered structure of the samples is evaluated using ATR-FTIR analysis. As shown in Figure 4, compared to the native group, the starch-lipid complexes exhibit strong absorption peaks at 2921, 2850, and 1700 cm^−1^, which correspond the stretching vibrations of -CH_2_ and -CH groups, the vibrations of -CH_2_ and -CH_3_ groups of the fatty acid carbon chains, and the vibrations of the -C=O group from fatty acids, respectively. Since most of the free fatty acids has been washed off after complexation reaction, the absorption peak around 1700 cm^−1^ is an indication of complex formation. This is consistent with the findings of Marinopoulou, et al. [41], in which an absorption peak at 1715 cm^−1^ was observed for starch-lipid complexes.

The spectrum in the fingerprint wavenumber range of 800–1200 cm^−1^ (Figure 4B) is further analyzed to characterize the crystalline and amorphous structures of the starch samples. Specifically, peak at 995 cm^−1^ is related to the number of hydrogen bonds in the short-range structure of the starch samples, while the peaks at 1022 cm^−1^ and 1047 cm^−1^ correspond to the amorphous and crystalline regions of the starch samples, respectively [42]. The ratio of these peaks provides insight into the short-range ordered structural characteristics. As shown in Table 2, compared to the control group (US-0-0), all ultrasonic-treated samples exhibit lower values in the absorbance ratio of the wavenumbers 1022 cm^−1^/995 cm^−1^. This indicates an increase in the number of hydrogen bonds in the short-range order, suggesting that more starch-lipid complexes are formed by stacking and associating to each other with enhanced hydrogen bonding, which results in a lower ratio. As the intensity of the ultrasound increases, the ratio decreases further, indicating that higher ultrasound intensity promotes the formation of these complexes. On the other hand, the absorbance ratio of the wavenumbers 1047 cm^−1^/1022 cm^−1^ decreases in the native group after gelatinization, likely due to the destruction of crystalline regions caused by the gelatinization process. After forming the complexes, the values show no clear trend. This lack of trend may be due to the high-temperature conditions during complex formation, combined with the non-uniform disruption of starch paste by ultrasound, leading to uneven degradation of both crystalline and amorphous regions on the surface.

### 3.4. Rapid-Visco Analysis (RVA)

**Figure 5:**
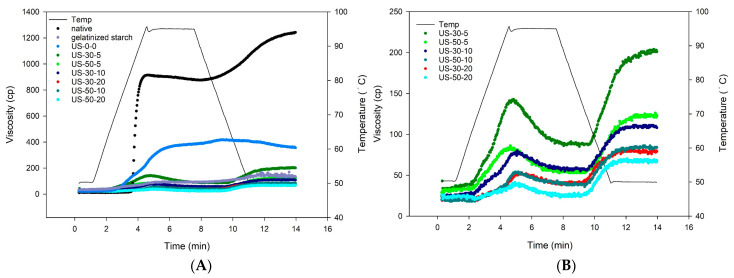
RVA spectra of (**A**) all sample groups and (**B**) ultrasonic-assisted water caltrop starch-lipid complex groups. Sample codes are the same as described in Figure 3.

Figure 5 shows the rapid-visco viscosity profiles of samples during continuous heating/cooling process, and the corresponding typical RVA parameters, including pasting temperature and peak, holding, breakdown, final and setback viscosities, are shown in Table 3. When measuring the viscosity of starch under excess water and high temperature, moisture enters the amorphous regions of the starch granules from the outside, compressing the crystalline regions. The moisture then interacts with the starch molecular chains inside the granules, forming a three-dimensional network structure that retains moisture and increases viscosity by creating a starch paste [43]. When the temperature decreases, the number of hydrogen bonds between starch chains increases, causing the molecules to aggregate into a gel and further increasing viscosity, as shown in the native water caltrop starch group in Figure 3. On the other hand, for the gelatinized starch group, structural disintegration during sample preparation leads to a decrease in viscosity.

As compared to the gelatinized starch group, the viscosity and pasting temperature of the control starch-fatty acid complex group without ultrasonic treatment (US-0-0) increase significantly, suggesting the formation of V-type crystalline structures. However, ultrasound pretreatment significantly decreases the viscosity of the starch-lipid complex group as compared to the control complex group. It is hypothesized that ultrasound affects the structural integrity of starch paste, breaking down the leached-out amylose molecules and even partially degrading the amylopectin molecules, which reduces the overall molecular weight of the sample and thus decreases viscosity. With a fixed amplitude, increasing the ultrasound duration results in significantly lower viscosity for both ultrasonic-treated groups with 30% and 50% amplitudes. Similarly, with a fixed treatment duration, as the ultrasound amplitude increases, the viscosity decreases accordingly, although the extent of viscosity reduction is less obvious as compared to groups with varying treatment durations.

### 3.5. DSC

**Figure 6:**
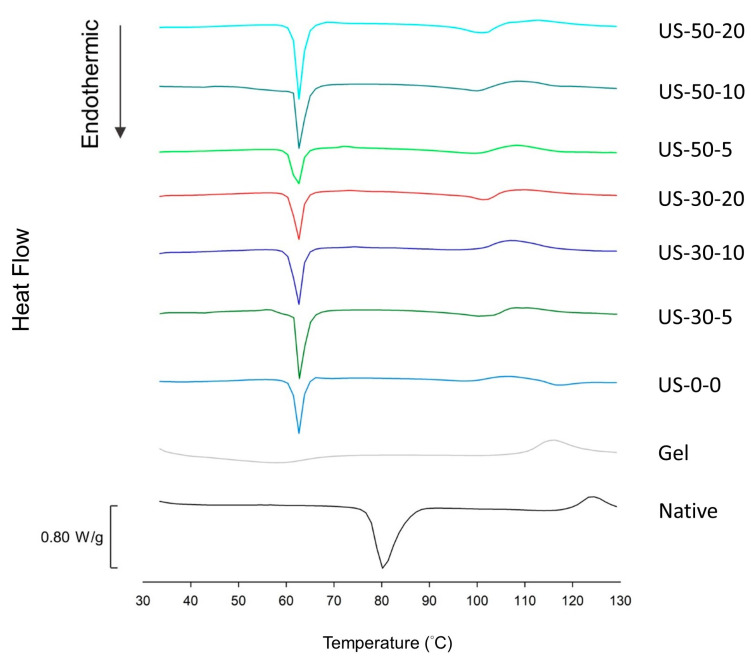
DSC spectra of native, gelatinized water caltrop starch and ultrasonic-assisted water caltrop starch-lipid complexes during heating. Sample codes are the same as described in Figure 3.

The DSC thermograms of the samples are presented in Figure 6, and the analysis of onset temperature (T_o_), peak temperature (T_p_), end set temperature (T_e_), and transition enthalpy (∆H) of the characteristic peaks are summarized in Table 4 for transitions below 90 °C, and Table 5 for transitions above 90 °C, respectively. As shown in Figure 6 and Table 4, the native water caltrop starch shows an endothermic peak at 76.98 °C, corresponding to its gelatinization temperature. For the gelatinized water caltrop starch, no peaks are observed, indicating the near-complete destruction of its internal structure during gelatinization. This result aligns with the X-ray diffraction data, which also show the loss of the starch’s crystalline structure. For the starch-lipid complexes, the first endothermic peak appears between 60.52 °C and 61.27 °C, corresponding to the melting temperature of free palmitic acid. This observation is consistent with the findings of Chao, et al. [44], suggesting that despite ethanol washing, small amounts of free fatty acids may remain in the complex samples. Furthermore, as shown in Figure 6 and Table 5, as the temperature goes higher than 90 °C, the control starch-lipid complex group (US-0-0) shows another two endothermic peaks above 90 °C, specifically between 93.07–103.97 °C and 112.56–122.00 °C, corresponding to Type I and Type II of V-type starch-lipid complexes, respectively [3]. The different characteristic temperature range may be related to the different structural arrangement [3]. Type I complex is thought to have less-ordered structure related to the randomly distributed single intrahelical starch-lipid complex and short-chain lamellae-like semi-crystalline structure. Type II complex is thought to have more ordered long-chain lamellae-like semi-crystalline structure. In contrast, only one endothermic peak above 90 °C is observed for the ultrasouic-treated samples, specifically between 93 °C and 105 °C, indicating the disappearance of the Type II complex after ultrasound treatment. Additionally, as the ultrasound intensity increases, the transition enthalpy (∆H) rises significantly. This increase in enthalpy (∆H) is attributed to the ultrasonic-assisted treatment, which breaks down amylose into smaller segments and partially disrupts amylopectin, releasing more short segments of pseudo-amylose. These short amylose molecules then interact with the guest molecule, palmitic acid, leading to the formation of more complexes and thus increasing the enthalpy (∆H). This result aligns with the ATR-FTIR data. Kong et al. [45] and Shogren et al. [46] also noted that longer amylose-fatty acid complexes align more precisely during stacking, resulting in raising the dissociation temperature. Similarly, long amylose chains are more likely to form Type II complexes, while the ultrasonic-modified amylose chains tend to form more Type I complexes.

### 3.6. Complexation Efficiency

Complex index is generally an indication of the complexation efficiency between fatty acids and starch, as during the measurement, the added iodine solution will react with the free or non-complexed amylose to generate an absorbance signal at 690 nm. The principle holds if the amount of amylose segments of the sample that could react with iodine is essentially the same as that of the reference (gelatinized starch), such as in the case of the control complex group (US-0-0) without ultrasound pretreatment. However, for the ultrasound-treated groups in this study, ultrasound is applied prior to complexation. The cavitation effect may lead to significant breakdown of amylose and amylopectin and increased release of apparent amylose from long segments of amylopectin. These amylose fragments if with appropriate DP may contribute to the formation of more starch-lipid complexes, however, still remain some un-complexed amylose and too-short amylose chain that cannot complex with fatty acids, but may complex with iodine, leading to a significant increase in absorbance and decrease in CI values (Table S1). As a result, direct comparison of the CI to evaluate the complexation efficiency between groups treated with different ultrasound intensities may not be appropriate. In fact, the decreased CI value with stronger ultrasound treatment is an indirect evidence qualitatively, demonstrating that ultrasound treatment can generate more short amylose segments and some long segments of amylopectin, and enhances the overall efficiency of complex formation, which aligns with the XRD and ATR-FTIR results.

### 3.7. Mechanism of Ultrasonic-Assisted Treatment on the Formation of Starch Lipid Complexes

The formation of crystalline starch-lipid V-complex requires several critical steps. During the complex formation process, starch must be solubilized in an aqueous solvent or heated to a sufficiently high temperature with excess water to disrupt the native helical segments. The presence of guest molecules induces a conformation change of the flexible random coil segments of starch, and associate into molecular-included helical complexes under an appropriate temperature higher than the crystallization temperature for a sufficient period of time, depending on the type and concentration of fatty acid and the DP of the flexible random coil segments of starch [9]. The subsequent crystallization process would influence the perfection of nucleation and crystal growth, resulting in allomorphs with different crystalline characteristics.

**Figure 7:**
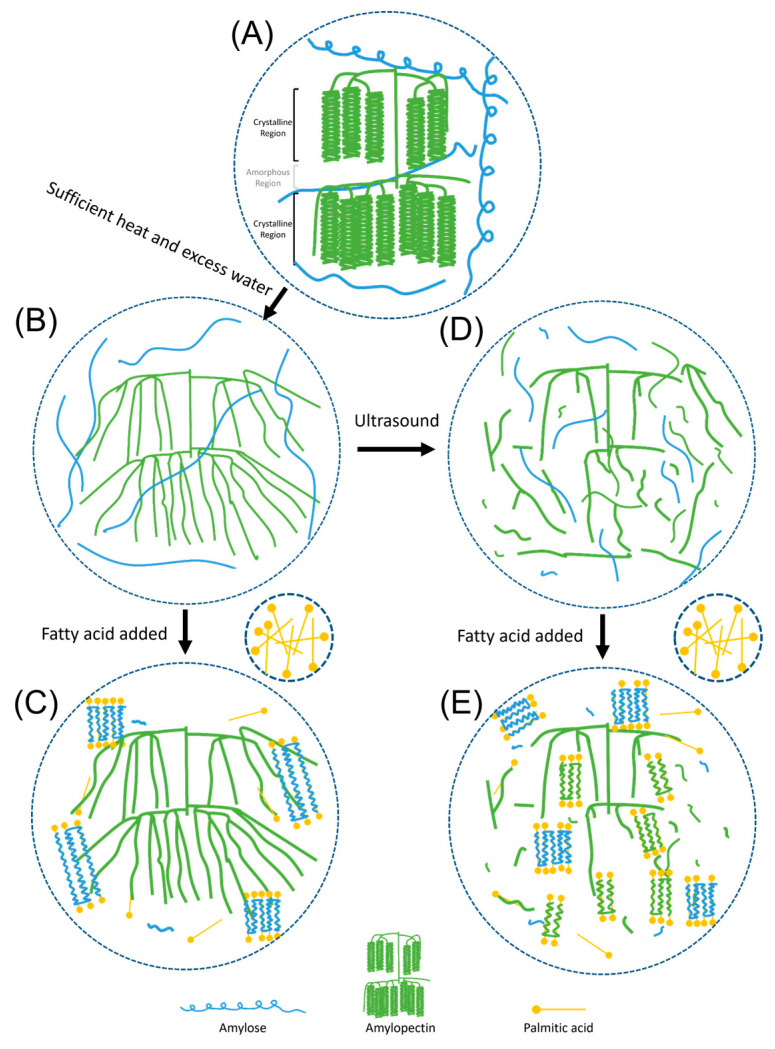
Schematic diagram of starch-lipid complex formation with and without ultrasonic-assisted pretreatment. (**A**) Native water caltrop starch, (**B**) Gelatinized water caltrop starch, (**C**) Starch-lipid complex without ultrasound pretreatment, (**D**) Ultrasonic-treated gelatinized starch, (**E**) Starch-lipid complex with ultrasound pretreatment.

Ultrasonic-assisted treatment significantly impacts the formation of these complexes. As illustrated in Figure 7, when starch undergoes swelling and gelatinization process due to sufficient heat and excess water, the crystalline and amorphous regions within the starch granules unravel, releasing amylose and disrupting the double helices of amylopectin, resulting in an expanded and swollen starch granule structure. These amylose molecules, varying in size and length, along with a minimal amount of amylopectin, complex with palmitic acid, stack and organize into long-chain Type II and short-chain Type I starch-lipid complexes.

In contrast, ultrasound treatment induces cavitation effects, causing non-specific physical disruption of the gelatinized starch, resulting in a polydisperse distribution of various branched maltodextrins. Though the residual branching point will probably hinder the ability to form stable single helices with lipids and crystallize, the prolonged ultrasound treatment with higher intensity may also lead to significant breakdown of amylose and amylopectin and increased release of apparent amylose from long segments of amylopectin. Since the shorter chain length and short branched chains can only form inclusion complexes with lower dissociation temperatures [45], primarily short-chain starch-lipid complexes (Type I of V-complex crystalline structure) is formed. This kind of mechanism is somewhat akin to the mechanism involving debranching enzymes [47], except that ultrasound-induced disruption is non-specific, generating a greater abundance of smaller, shorter amylose chains. Notably, these amylose chains vary in length, and some of the excessively short chains fail to participate in the complexation reaction due to their low degree of polymerization (DP). Overly intense ultrasound treatment could decrease the effectiveness of complex formation [2,20], and appropriate ultrasound intensity is a crucial factor in the successful formation of starch-lipid complexes.

## 4. Conclusions

This study explores the formation of water caltrop starch-lipid complexes using ultrasonic-assisted treatment. Results from polarized light microscopy and scanning electron microscopy reveal the polarized characteristics and morphological structure of the complexes. The V-type characteristics from XRD analysis further confirm the successful formation of water caltrop starch-lipid complexes using ultrasonic-assisted treatment. ATR-FTIR and XRD analyses show that as ultrasound intensity increase, both short-range and long-range order display upward trends. DSC comparison between untreated and ultrasound-treated complexes reveal the disappearance of Type II starch-lipid complexes by ultrasound treatment. Additionally, as ultrasound intensity increase, the enthalpy of Type I starch-lipid complexes also increases, indicating the breakdown of amylopectin and amylose, followed by their interaction with fatty acids, lead to the formation of more Type I complexes, which in turn enhances bonding and crystalline structures between the complexes. This result also corresponds with the RVA findings, which show a decrease in viscosity possibly by lower molecular weight. This study offers a comprehensive analysis of the effects of ultrasound treatment on amylose and amylopectin to form starch-lipid complexes. It contributes to future research on the impact of ultrasound on starch and the production of Type I starch-lipid complexes, while also providing a deeper understanding of the mechanism involved in starch-lipid complex formation. However, the digestive hydrolysis of Type V resistant starch and its impact on the functionality of processed foods depend not only on the amount of complex formed, but also the structural characteristics of the complex. Further investigation on the short-range order of the developed complex by nuclear magnetic resonance (NMR), changes in chain length distribution, functional properties, as well as in-vitro and in-vivo digestibility, would be valuable for gaining a deeper understanding of the molecular docking mechanisms and potential applications in the food industries.

## Figures and Tables

**Table 1:** The long-range ordered structural characteristics of water caltrop starch, gelatinized water caltrop starch and ultrasonic-assisted water caltrop starch-lipid complexes by XRD analysis ^1–3^.

Sample Code ^1^	Relative Crystallinity (%)	Crystalline Type
Native	46.7 ± 0.6 ^a^	A type
Gelatinized Starch	3.2 ± 0.4 ^f^	- ^2^
US-0-0	20.9 ± 0.6 ^e^	V-type
US-30-5	24.6 ± 0.4 ^d^	V-type
US-30-10	26.1 ± 0.5 ^c^	V-type
US-30-20	28.7 ± 0.2 ^b^	V-type
US-50-5	26.1 ± 0.6 ^c^	V-type
US-50-10	26.7 ± 0.2 ^c^	V-type
US-50-20	27.9 ± 0.3 ^b^	V-type

^1^ Sample codes are the same as described in Figure 3. ^2^ “-” indicates the type of crystalline structure cannot be determined. ^3^ Values in the same column with different superscripts are significantly different (*p* < 0.05).

**Table 2:** The short-range ordered structural characteristics of water caltrop starch, gelatinized water caltrop starch and ultrasonic-assisted water caltrop starch-lipid complexes by ATR−FTIR analysis ^1,2^.

Sample Code ^1^	R_1047 cm_^−1^_/1022 cm_^−1^	R_1022 cm_^−1^_/995 cm_^−1^
Native	0.725 ± 0.009 ^a^	0.783 ± 0.001 ^e^
Gelatinized Starch	0.696 ± 0.008 ^b^	0.890 ± 0.003 ^d^
US-0-0	0.674 ± 0.014 ^c^	0.919 ± 0.000 ^a^
US-30-5	0.674 ± 0.006 ^c^	0.917 ± 0.005 ^ab^
US-30-10	0.680 ± 0.002 ^bc^	0.910 ± 0.009 ^abc^
US-30-20	0.682 ± 0.008 ^bc^	0.898 ± 0.007 ^cd^
US-50-5	0.673 ± 0.008 ^c^	0.906 ± 0.003 ^bc^
US-50-10	0.683 ± 0.011 ^bc^	0.906 ± 0.002 ^bc^
US-50-20	0.6659 ± 0.011 ^c^	0.904 ± 0.005 ^c^

^1^ Sample codes are the same as described in Figure 3. ^2^ Each data is expressed as the mean ± standard deviation. Values in the same column with different superscripts are significantly different (*p* < 0.05).

**Table 3:** Rapid viscosity analysis (RVA) results of water caltrop starch, gelatinized water caltrop starch and ultrasonic-assisted water caltrop starch-lipid complexes ^1,2^.

Sample Code ^1^	Pasting Temperature (°C)	Peak Viscosity (cP)	Holding Viscosity (cP)	Breakdown Viscosity (cP)	Final Viscosity (cP)	Setback Viscosity (cP)
Native	83.0 ± 0.5 ^a^	900.0 ± 13.5 ^a^	850.7 ± 22.1 ^a^	49.3 ± 10.3 ^b^	1203.3 ± 32.7 ^a^	352.7 ± 10.7 ^a^
Gelatinized Starch	63.9 ± 0.7 ^d^	108.0 ± 11.3 ^d^	104.7 ± 11.9 ^c^	3.3 ± 2.1 ^e^	156.7 ± 6.8 ^d^	52.0 ± 5.2 ^d^
US-0-0	66.1 ± 0.7 ^b^	425.7 ± 1.5 ^b^	420.7 ± 2.5 ^b^	1.7 ± 2.3 ^e^	390.0 ± 7.9 ^b^	−30.7 ± 5.5 ^f^
US-30-5	64.8 ± 0.7 ^cd^	154.0 ± 13.5 ^c^	90.0 ± 3.6 ^c^	64.0 ± 1.1 ^a^	201.0 ± 0.0 ^c^	111.0 ± 3.6 ^b^
US-30-10	64.3 ± 0.6 ^cd^	84.7 ± 4.2 ^e^	60.0 ± 4.4 ^d^	24.7 ± 0.6 ^c^	115.7 ± 6.8 ^e^	55.7 ± 2.5 ^d^
US-30-20	66.1 ± 0.3 ^b^	50.3 ± 2.3 ^fg^	41.3 ± 2.9 ^e^	9.0 ± 5.2 ^e^	73.7 ± 5.0 ^f^	32.3 ± 7.8 ^e^
US-50-5	65.4 ± 0.9 ^bc^	111.7 ± 0.6 ^d^	67.3 ± 3.1 ^d^	44.3 ± 2.5 ^b^	152.7 ± 0.6 ^d^	85.3 ± 2.5 ^c^
US-50-10	65.3 ± 0.5 ^bc^	57.7 ± 3.5 ^f^	37.0 ± 1.0 ^e^	20.7 ± 4.5 ^c^	90.0 ± 7.2 ^f^	51.0 ± 4.6 ^d^
US-50-20	66.0 ± 0.3 ^b^	39.7 ± 1.2 ^g^	32.0 ± 1.2 ^e^	7.3 ± 1.2 ^e^	70.0 ± 1.7 ^f^	37.7 ± 0.6 ^e^

^1^ Sample codes are the same as described in Figure 3. ^2^ Each data is expressed as the mean ± standard deviation. Values in the same column with different superscripts are significantly different (*p* < 0.05).

**Table 4:** Thermal parameters of native, gelatinized water caltrop starch and ultrasonic-assisted water caltrop starch-lipid complexes from the endothermic peaks below 90 °C in DSC ^1–4^.

Sample Code ^1^	Native	Gelatinized Starch	US-0-0	US-30-5	US-30-10	US-30-20	US-50-5	US-50-10	US-50-20
T_o_ (°C)	76.98 ± 0.06 ^a^	-	61.06 ± 0.33 ^b^	60.98 ± 0.48 ^b^	60.52 ± 0.12 ^b^	60.91 ± 0.54 ^b^	60.62 ± 0.18 ^b^	61.27 ± 0.02 ^b^	61.17 ± 0.38 ^b^
T_p_ (°C)	79.95 ± 0.12 ^a^	-	62.23 ± 0.23 ^b^	62.23 ± 0.21 ^b^	61.97 ± 0.12 ^b^	62.22 ± 0.46 ^b^	62.07 ± 0.23 ^b^	62.46 ± 0.13 ^b^	62.36 ± 0.23 ^b^
T_e_ (°C)	84.51 ± 0.04 ^a^	-	64.05 ± 0.39 ^b^	64.15 ± 0.30 ^b^	63.73 ± 0.16 ^b^	63.98 ± 0.51 ^b^	63.97 ± 0.28 ^b^	64.37 ± 0.11 ^b^	64.25 ± 0.36 ^b^
T_e_-T_o_ (°C)	7.54 ± 0.11 ^a^	-	2.99 ± 0.06 ^c^	3.17 ± 0.18 ^bc^	3.21 ± 0.04 ^bc^	3.08 ± 0.03 ^c^	3.34 ± 0.10 ^b^	3.10 ± 0.14 ^c^	3.09 ± 0.02 ^c^
∆H (J/g)	23.88 ± 0.56 ^a^	-	10.15 ± 0.10 ^c^	10.87 ± 2.62 ^bc^	11.19 ± 0.59 ^bc^	10.96 ± 1.97 ^bc^	9.02 ± 0.28 ^c^	11.67 ± 1.65 ^bc^	13.74 ± 0.13 ^b^

^1^ Sample codes are the same as described in Figure 3. ^2^ T_o_, T_p_, T_e_, T_e_-T_o_ and ∆H indicate onset temperature, peak temperature, end set temperature, transition temperature range and transition enthalpy, respectively. ^3^ “-” indicates that no peak was observed. ^4^ Each data is expressed as the mean ± standard deviation. Values in the same column with different superscripts are significantly different (*p* < 0.05).

**Table 5:** Thermal parameters of native, gelatinized water caltrop starch and ultrasonic-assisted water caltrop starch-lipid complexes from the endothermic peaks above 90 °C in DSC ^1–4^.

Sample Code ^1^	Native	Gelatinized Starch	US-0-0	US-30-5	US-30-10	US-30-20	US-50-5	US-50-10	US-50-20
Peak I									
T_o_ (°C)	-	-	93.07 ± 0.41 ^c^	96.24 ± 0.15 ^a^	95.56 ± 0.91 ^ab^	96.24 ± 0.29 ^a^	96.02 ± 1.42 ^a^	95.76 ± 0.83 ^ab^	93.92 ± 0.49 ^bc^
T_p_ (°C)	-	-	98.75 ± 0.01 ^c^	101.07 ± 0.25 ^ab^	100.81 ± 0.58 ^ab^	101.56 ± 0.47 ^a^	101.06 ± 0.70 ^ab^	100.14 ± 0.58 ^b^	100.40 ± 0.47 ^ab^
T_e_ (°C)	-	-	103.97 ± 0.79 ^b^	104.88 ± 0.25 ^ab^	105.69 ± 0.33 ^a^	105.00 ± 0.00 ^ab^	104.49 ± 0.74 ^ab^	104.45 ± 0.47 ^ab^	105.27 ± 0.52 ^ab^
T_e_-T_o_ (°C)	-	-	10.90 ± 1.20 ^a^	8.64 ± 0.40 ^b^	10.13 ± 0.57 ^ab^	8.77 ± 0.29 ^b^	8.47 ± 0.68 ^b^	8.69 ± 1.30 ^b^	11.35 ± 1.01 ^a^
∆H (J/g)	-	-	1.43 ± 0.35 ^c^	1.83 ± 0.21 ^c^	1.51 ± 0.88 ^c^	3.73 ± 0.21 ^b^	2.32 ± 0.05 ^c^	3.46 ± 0.65 ^b^	5.08 ± 0.01 ^a^
Peak II									
T_o_ (°C)	-	-	112.56 ± 0.06	-	-	-	-	-	-
T_p_ (°C)	-	-	116.37 ± 0.47	-	-	-	-	-	-
T_e_ (°C)	-	-	122.00 ± 1.32	-	-	-	-	-	-
T_e_-T_o_ (°C)	-	-	9.45 ± 1.38	-	-	-	-	-	-
∆H (J/g)	-	-	1.95 ± 0.24	-	-	-	-	-	-

^1^ Sample codes are the same as described in Figure 3. ^2^ T_o_, T_p_, T_e_, T_e_-T_o_ and ∆H indicate onset temperature, peak temperature, end set temperature, transition temperature range and transition enthalpy, respectively. ^3^ “-” indicates that no peak was observed. ^4^ Each data is expressed as the mean ± standard deviation. Values in the same column with different superscripts are significantly different (*p* < 0.05).

## Data Availability

The data presented in this study are available on request from the corresponding author. The data are not publicly available due to ethical restriction and the intellectual property issue.

## Supplementary Materials

The following supporting information can be downloaded at: https://www.mdpi.com/article/10.3390/foods14020240/s1, Table S1: Complex index of starch-lipid complex using different intensity of ultrasonic treatment.
