# Supplementary material for: Impact of Ultrasonic-Assisted Preparation of Water Caltrop Starch–Lipid Complex: Structural and Physicochemical Properties
Source: Foods. 2025 Jan 14;14(2):240. doi: 10.3390/foods14020240 (PMC11765270; doi:10.3390/foods14020240)
Supplement: Supplementary file 1 [file foods-14-00240-s001.zip › foods-3390939-supplementary.pdf]

Table S1. Complex index of starch-lipid complex using different intensity of ultrasonic treatment.[1-2]

**Sample code**[1]	**Complex index (%)**
US-0-0	80.76±0.00[a]
US-30-1	71.88±0.32[b]
US-30-5	30.74±1.52[c]
US-30-10	8.17±2.58[d]
US-50-30	-30.22±3.32[e]

[1] Sample codes are designated as follows. "US-0-0" denotes the starch-lipid complex without ultrasonic treatment; "US-30-1", "US-30-5", and "US-30-10" denote samples treated with 30% ultrasonic amplitude for 5, 10, and 20 minutes, respectively; and "US-50-30" denotes samples treated with 50% ultrasonic amplitude for 30 minutes.
[2] Values in the same column with different superscripts are significantly different ($p < 0.05$).
